# Effective Way of Studying and Learning in Medical School

**DOI:** 10.31729/jnma.5258

**Published:** 2020-11-30

**Authors:** Shreya Niroula, Aliska Niroula

**Affiliations:** 1 Lumbini Medical College and Teaching Hospital, Prabhas, Palpa, Nepal; 2 Kathmandu Medical College and Teaching Hospital, Sinamangal, Kathmandu, Nepal

**Keywords:** *active recall*, *medical students*, *spaced repetition*, *study techniques*

## Abstract

Medical knowledge is growing. We struggle a lot to retain most of the studied information, but we forget it. Most of us are unaware of how to study effectively and improve memory. Understanding the neurobiology of learning and knowing some of the productive study techniques backed up by the researches can help us to study as well as learn effectively. This article may help medical students to study productively and motivate them to search for effective ways of studying actively.

The meaning of studying is to read and memorize facts to learn about a subject. Likewise the meaning of learning means to gain knowledge or skill by studying, practising, being taught or experiencing something. We are born with a very premature brain-probably the result of an evolutionary compromise. As our brain starts to develop, we start to learn every waking moment.

In medical schools, being able to study effectively is essential to cope with the vast amount of information and skills needed to be acquired as medical knowledge is growing. Despite an increase in depth and complexity of medical knowledge in the past decades, the time for medical education remains constant. Most of us are unaware of how to learn effectively and improve memory. We struggle a lot to retain most of the studied information, but we forget it. Understanding the neurobiology of learning and knowing some of the study techniques backed up by the researches can help us to study as well as learn effectively.

## NEUROBIOLOGY OF LEARNING

Neuroscientists have defined the various molecular signalling pathways within and between neurons that play a role in learning.^1^ The learning experience is stored as a persistent representation, moving from a transient working memory stage that has a relatively limited capacity and time frame to a more long-lasting and stable form of memory with larger capacity that is stored for future access.^2^ Learning leads to functional and structural changes in the interconnected cellular networks between neurons (synapses) at a variety of sites throughout the central nervous system.^3^ Recent studies have shown that these changes involve a variety of posttranslational modifications of proteins located in proximity to synaptic contacts that can enhance the strength of subsequent signals produced by a presynaptic nerve impulse (action potential) at the postsynaptic neuron.^4^ This type of experimental work provides a direct link between the spaced repetition of the information to be learned and the persistence of the changes in the nervous system that accompany such learning.^1^

## STUDY TECHNIQUES BASED ON RESEARCHES

According to Dunlosky et al,^5^ the techniques in order of utility test assessment are as follows:

### A. High Utility Assessments

- **Practise testing:** Self-testing or taking practise tests over to-be-learned materials. Taking regular tests or exams very useful to study effectively.
- **Distributed practise:** Implementing a schedule of practise that spreads out study activities over time. Making flashcards is very useful to apply the technique in real life. Anki flashcards^6^ can be a useful tool in this context.

### B. Moderate Utility Assessments

- **Interleaved practise:** Implementing a schedule of practise that mixes different kinds of problems, or a schedule of study that mixes different kinds of material, within a single study session.
- **Elaborative interrogation:** Generating an explanation for why an explicitly stated fact or concept is true.

### C. Low Utility Assessment

- **Self-explanation:** Explaining how new information is related to known information, or explaining steps taken during problem-solving.
- **Summarization:** Writing summaries of to-be-learned texts.
- **Highlighting/underlining:** Marking potentially important portions of to-be-learned materials while reading.
- **Keyword mnemonic:** Using keywords and mental imagery to associate verbal materials.
- **Imagery for text:** Attempting to form mental images of text materials while reading or listening.
- **Re-reading:** Restudying text material after an initial reading.

## IMPORTANCE OF PRACTICE TESTING AND DISTRIBUTED PRACTISE (SPACED REPETITION)

We have given numerous exams but most of us do not know the essence of the exams. Taking exams is an active and effective way of learning than studying the same thing repeatedly. It also helps in the retention of the information. Also, in case, we cannot answer a test question the retrieval attempts to enhance our future learning.^7^ Likewise, frequent classroom testing stimulates practise and review which gives students more opportunities for feedback on their work and has a positive influence on student study time.^8^ But, when the students study on their own, most of them repeatedly read their notes or textbook despite the limited benefits of this strategy. Only a few engage themselves in self-testing or retrieval practise while studying.^9^

## LEARNING IN MEDICAL SCHOOL

Learning in medical school comprises of two types of knowledge:

1. Factual or theoretical knowledge
2. Procedural or practical knowledge

Factual or conceptual knowledge covers “what” information, whereas procedural knowledge covers “how” and “why” information.^10^ We can become proficient in gaining factual knowledge by practise testing, spaced repetition or distributed practise and interleaved practise. Likewise, we can become proficient in gaining procedural knowledge by problem-based learning and self-regulated learning. Problem-based learning is not only about problem-solving, but rather it uses appropriate problems to increase knowledge and understanding by going through the triggers and discussing the cases among colleagues and supervisor in a focused group.^11^ There is diversity in the use of self-regulated learning strategies by medical students, which is linked to an individual (goal setting), contextual (time pressure, patient care and supervision) and social (supervisors and peers) factors.^12^

## WAYS FORWARD

Since early childhood, the majority of us were taught to study but we were barely taught about how to study effectively. Medical knowledge is vast, it is evolving now and then. Most of us find medical school to be very difficult because we are unaware of the ways of studying effectively. A lot of researches are being done in studying and learning techniques. We should update ourselves and actively search for new ways to study productively.

## Conflict of Interest

**None.**
